# Therapeutic impact of the ethyl acetate extract of *Tripterygium wilfordii *Hook F on nephritis in NZB/W F1 mice

**DOI:** 10.1186/ar1879

**Published:** 2006-01-03

**Authors:** Xuelian Tao, Fred Fan, Victoria Hoffmann, Nancy S Longo, Peter E Lipsky

**Affiliations:** 1 Autoimmunity Branch, National Institute of Arthritis and Musculoskeletal and Skin Diseases, National Institutes of Health, Rockville Pike, Bethesda, MD 20892, USA; 2 Office of Research Services Division of Veterinary Resources, National Institutes of Health, Rockville Pike, Bethesda, MD 20892, USA

## Abstract

This study was designed to examine the potential use of the ethyl acetate (EA) extract of *Tripterygium wilfordii *Hook F (TwHF), a Chinese herbal medicine, in the treatment of systemic lupus erythematosus. A total of 48 28-week-old female NZB/W F1 mice were randomly divided into three groups and orally administered vehicle or the EA extract of TwHF at 18.25 mg/kg (EA_low_) or 36.5 mg/kg (EA_high_) for 14 weeks. Proteinuria and serum anti-double-stranded (ds)DNA antibody titers were assayed before and after treatment. At the end of treatment, all animals were sacrificed and pathological changes in the kidneys were examined by observers blinded to the treatment regimens. Immunohistological studies were carried out on kidneys and spleens. At 28 weeks of age, proteinuria (>30 mg/dl) and anti-dsDNA antibodies were found in all mice in the three groups. Fourteen, sixteen and fifteen mice in the vehicle, EA_low _and EA_high _groups, respectively, completed at least four weeks of treatment. At the end of treatment, the mean proteinuria of the EA_low _and EA_high _groups was significantly less than that of the vehicle group and no different from proteinuria at the onset of treatment. Histological evidence of glomerulonephritis, glomerular deposition of IgG and complement 3 and cellular infiltration in the interstitium and perivascular regions were significantly less severe in the EA extract treated mice than in vehicle treated mice. Treatment with the EA extract significantly inhibited the progression of kidney disease in NZB/W F1 mice, though had no significant effect on the levels of anti-dsDNA antibody.

## Introduction

The Chinese anti-rheumatic remedy *Tripterygium wilfordii *Hook F (TwHF) has been reported to be effective in the treatment of a variety of autoimmune diseases, including rheumatoid arthritis (RA), systemic lupus erythematosus (SLE), and psoriasis [1,2]. The therapeutic benefit of TwHF preparations in patients with a variety of kidney diseases, including IgA nephropathy and Henoch-Schonlein purpura nephritis, has also been described [3-6]. Moreover, in several uncontrolled trials, improvement in clinical manifestations and laboratory abnormalities was observed in as many as 94% of SLE patients treated with a variety of TwHF preparations [7-10].

Different preparations of TwHF have been tested for their therapeutic effect in the MRL-*lpr*/*lpr *murine model of lupus. The TwHF preparations employed in these studies were crude extracts and their composition was not known. Therefore, it is difficult to assess the pharmacological impact of the material or to standardize the extract for further development. Gu and colleagues [11] found that a water extract of TwHF ameliorated glomerulonephritis and prolonged survival in MRL-*lpr*/*lpr *mice, but only when therapy was begun before disease onset. Consistent with this, Zhang and colleagues [12] reported improvement in survival, proteinuria, arthritis and lymphadenopathy in MRL-*lpr*/*lpr *mice treated with another TwHF preparation. However, no improvement in renal histology was noted. Although both of these studies suggest benefit in this model of murine lupus when treatment was begun before disease onset, the full extent of potential benefit was not established. Importantly, no evidence was provided that the extract of TwHF was beneficial as treatment after onset of autoimmune disease.

NZB/W F1 mice spontaneously develop autoantibodies against double-stranded (ds)DNA; these antibodies form immune complexes with dsDNA. Deposition of the immune complex in the kidney induces activation of the complement system, which consequently results in chronic glomerulonephritis, vasculitis and cellular infiltration in the interstitium of the kidney [13,14]. This animal model has been commonly used for screening of drugs for treatment of human SLE because of its similarities to human SLE in clinical, immunopathological, and genetic features [15-18]. Specifically, the high incidence of SLE-like disease, characterized by gender selectivity, chronic immune complex nephritis and high titers of anti-dsDNA antibody, makes it possible to evaluate efficacy of treatment easily in the NZB/W F1 mice. This animal model, however, had not yet been employed to assess the impact of the TwHF preparations.

An ethyl acetate (EA) extract of TwHF has been prepared and used for the first time in the United States in a controlled, double-blinded clinical trial of patients with RA [19,20]. Results from the trial showed significant therapeutic benefit and good tolerance in treated RA patients. The EA extract of TwHF has been studied in detail for its content of active components, namely the diterpenoids, triptolide and tripdiolide, as well as its *in vitro *and *in vivo *anti-inflammatory and immunosuppressive impact and toxicity [21-25]. Importantly, the EA extract of TwHF can be standardized by quantitatively assessing its content of active components, as well as with regard to efficacy and adverse events. To estimate the potential therapeutic effect of this standardized extract on patients with SLE, experiments with NZB/W F1 mice were undertaken. NZB/W F1 mice with established nephritis were treated orally with vehicle only or the EA extract for a total of 14 weeks beginning at 28 weeks of life. Kidney disease significantly worsened in more than 90% of the mice treated with vehicle. In contrast, kidney disease was improved or controlled in NZB/W F1 mice treated with either a low or a high dose of the EA extract, suggesting an important therapeutic effect of this standardized extract in this animal model of lupus.

## Materials and methods

### The ethyl acetate extract

The EA extract was prepared as described and analyzed for its content of triptolide and tripdiolide, which are responsible for up to 90% of the bioactivity of the EA extract [21]. In addition, the EA extract was assessed for the amount that caused death of 50% of treated C57BL/6j mice (LD50) as described. Briefly, plant material for extraction was assessed by HPLC for its content of diterpenoids, after which selected peeled roots of TwHF were ground and extracted with EA. The EA extract was concentrated to dryness and ground to a fine powder. The final preparation contained 0.77 μg/mg of triptolide and 0.44 μg/mg of tripdiolide.

### Animals and treatment regimens

Eight week old female NZB/WF1/J mice were purchased from Jackson Laboratory (Bar Harbor, ME, USA) and maintained in a conventional animal housing facility throughout the study. At 28 weeks of age, the animals were randomly divided into three treatment groups (vehicle, EA_low _and EA _high_) and orally administered vehicle (2% dimethyl sulfoxide/ 5% Tween-20 in water), the EA extract of TwHF at 18.25 mg/kg (equivalent to 1/20 of the LD50) or the EA extract of TwHF at 36.5 mg/kg (equivalent to 1/10 of the LD50), respectively. This equated to 28.1 μg/kg of triptolide and 16 μg/kg of tripdiolide. The EA extract was dissolved in vehicle solution to obtain an appropriate concentration for oral administration (about 0.4 ml per animal per dose). Based on the information obtained from a previous study demonstrating that the half life of triptolide was 6.2 h after oral administration, treatment was given daily, 5 days a week from Monday to Friday for a total of 14 weeks. Body weight was monitored weekly. If an animal lost more than 15% of body weight, treatment was terminated and the animal euthanized. Otherwise, mice were sacrificed after 14 weeks of treatment. Blood, kidneys and spleen were collected from all mice, including those dying before completion of treatment and those completing the treatment protocol.

The study proposal (A-002-03-03) has been approved and all procedures monitored by the Animal Care and Use Committee (ACUC) in the Intramural Research Program of National Institutes of Health.

### Urine collection and proteinuria assay

Urine from individual mice was collected biweekly from the age of 24 weeks using metabolic cages. Proteinuria was tested by dipstrip (Chemstrip, Roche (Indianapolis, IN 46256 USA) and semiquantified as 0, ± (0 to 30 mg/dl), + (30 to 100 mg/dl), ++ (100 to 500 mg/dl) and +++ (>500 mg/dl). Proteinuria was also analyzed by spectrophotometer using a bicinchoninic acid based BCA protein assay kit (Pierce, Rockford, IL, USA) and standardized with BSA. In the entire study course, proteinuria was measured first with the urinary analysis strips (Chemstick) followed by the spectrophotometer. It was found that results obtained from the Chemstick correlated with those by spectrophotometer. Since proteinuria was quantified more accurately, data generated using the spectrophotometer were used to plot the figures.

### Assay for blood urea nitrogen

Serum blood urea nitrogen (BUN) was determined by the NIH Diagnostic and Research Service Branch, the laboratory of the Veterinary Resources Program.

### ELISA for anti-dsDNA antibodies [26]

EIA/RIA plates (96-well; Corning Inc., Corning, NY, 14831, USA) were coated with 50 μl of 10 μg/ml type XV calf thymus DNA (Sigma, St Louis, MO 63103, USA) overnight at 4°C. After washing, the plates were blocked with 10% BSA/PBS. The plates were incubated with diluted serum (first dilution of 1:20 followed by serial 3-fold dilutions) for 2 h at room temperature. After washing with 0.5% Tween-20/PBS, the plates were incubated with alkaline phosphatase conjugated goat anti-mouse IgG (Fab) for 1 h followed by the alkaline phosphatase substrate *p*-nitrophenyl phosphate. The reaction was stopped by addition of 25 μl of 5N NaOH solution. The plates were assayed with a microplate spectrophotometer (Bio-Tek Instrument Inc., Winooski, VT 05404-0998, USA) at 405 nm. Sera from 10 C57BL/6j mice were employed as normal controls.

### Pathological study of kidneys

Kidneys and spleens were harvested from the mice after spontaneous death or euthanasia. One-half of the kidney from each mouse was immersed in TBS tissue freezing medium (Triangle Biomedical Sciences, Durham, NC, 27705, USA) and snap frozen in methanol-dry ice. The other half of the freshly harvested kidney was fixed in buffered 10% formalin (Fisher Scientific, Pittsburgh, PA 15275-9952, USA) and embedded in paraffin blocks (Sugipath, Richmond, IL 60071, USA). Seven micron thick sections were cut and stained with hematoxylin and eosin (Histoserv, Germantown, MD 20874-1202, USA). Sections were graded semi-quantitatively by two veterinary pathologists for glomerular, interstitial and vascular lesions. The grading scheme, a modification of the method reported by Chan and colleagues [27], is shown in Table 1.

### Flow cytometry analysis of spleen cells

Mononuclear cells separated from spleens of the NZB/W F1 mice were stained for B cells, T cells and dendritic cells using Allophycocyanin (APC) conjugated rat anti-mouse CD19, fluorescein isothiocyanate (FITC) conjugated rat anti-mouse CD3 and Phycoerythrin (PE) conjugated rat anti-mouse CD11c antibodies, respectively (BD Biosciences, Pharmingen, San Diego, CA 92121-8995, USA After staining, the cells were analyzed by flow cytometry using a FACSCalibur (Becton Dickson, San Jose, CA, USA) along with CellQuest software (BD Biosciences, San Jose, CA 95131, USA) for data acquisition and analysis.

### Staining of IgG and complement 3 in kidneys

For determination of IgG deposition, kidney cryosections were stained using FITC conjugated goat anti-mouse IgG antibody (Cappel, MP Biomedical Inc., Aurora, OH 44202, USA). For determination of complement 3 (C3) deposition, kidney cryosections were stained with horseradish peroxidase conjugated goat anti-mouse C3 antibody (MP Biomedical Inc.) followed by color development with the VIP substrate kit (Vector Laboratories, Burlingame, CA 94010, USA). The slides were counter-stained with hematoxylin (Vector Laboratories).

### Fluorescent staining of lymphoid cells in spleens and kidneys

Frozen sections were used for examination of lymphoid cell subsets in spleen and cell infiltration in kidneys. For determination of CD3^+ ^T cells, the frozen sections were first blocked with 10% goat serum and stained with rat anti-mouse CD3^+ ^antibody (BD Pharmingen) followed by succinimidyl ester-labeled goat anti-rat IgG antibody (Alexa Fluor 568, Molecular Probes, Eugene, OR 9402-2209, USA). For determination of CD11c^+ ^cells, the cryosection was blocked with 10% mouse serum followed by sequential staining with hamster anti-mouse CD11c (BD Pharmingen) and FITC conjugated mouse anti-hamster IgG (BD Pharmingen). For identification of B cells, cryosections were directly stained with FITC conjugated rat anti-mouse IgD (BD Pharmingen).

Images were captured using a Hamamatsu camera system connected to a microscope (Leica DMRB) along with the IPLab Scientific Image Processing software (Scanalytic Inc., Rockville, MD 20850, USA). Two color photos were developed using the software Adobe Photoshop version 7.

### Statistical analysis

An intention-to-treat analysis was carried out, including all NZB/W F1 mice that completed at least four weeks of the treatment. All statistical tests were two sided. Comparison of the mean values of individual variables measured at each time to the corresponding base-line values for mice of the same group was carried out using the Student's *t *test. The Kruskal-Wallis test was employed to compare each variable between groups before and after treatment. For proteinuria data, a last-observation-carried-forward approach was used for the mice that died before the end of study.

## Results

### Outcome

Initially, 16 mice were included in each group. One mouse from each group had severe proteinuria at the beginning of treatment and was dead within four weeks of starting the treatment. One animal of the vehicle group unexpectedly died of acute pulmonary edema caused by impropriate gavage. Data from these four mice were excluded from analysis of treatment efficacy. One animal from each group completed more than four weeks of treatment, but died before the end of the treatment course after development of severe proteinuria and elevated BUN, suggesting disease related death. These mice were included in the analysis. Two mice from the EA_low _group were euthanized at the eighth and ninth week of the EA treatment because of weight loss reaching 15% of baseline. Despite that, there was no significant difference in the mean body weight between the three groups either before or after treatment (42.3 g, 41.6 g and 40.8 g before starting treatment and 40.4 g, 39.5 g and 40.2 g after treatment for vehicle, EA_low _and EA_high _groups, respectively). These mice were also included in the analysis.

### Therapeutic effect of the EA extract on proteinuria and renal function

**Figure 1:**
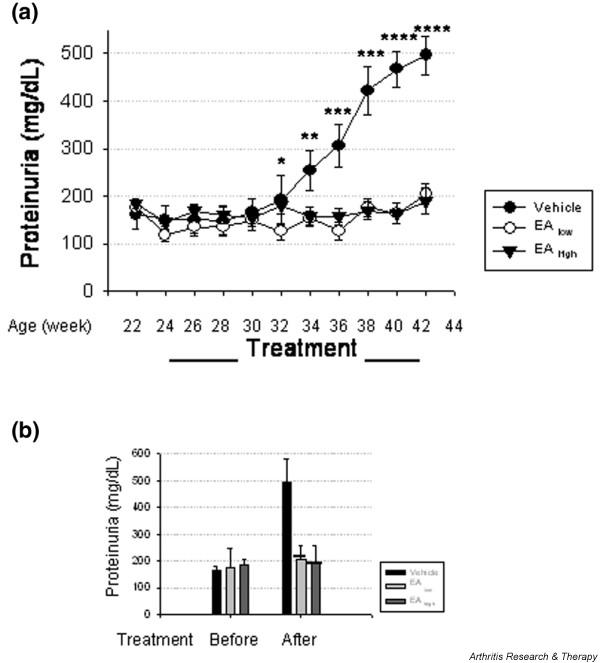
Impact of treatment with *Tripterygium wilfordii *Hook F on proteinuria in NZB/W F1 mice. Proteinuria was determined by spectrophotometry as described in Materials and methods. Data are the means ± standard error of the mean of each group. Numbers in parentheses indicate the animals examined. **(a) **Changes in proteinuria in NZB/WF1 mice during the treatment course. Statistical analysis was done for proteinuria measured before and at each time point after treatment for each group. Proteinuria increased significantly in the mice of the vehicle group (*p < 0.05; ***p < 0.001; ****p < 0.0001; before treatment verses after treatment). **(b) **Proteinuria was compared between groups before and after treatment. No significant difference was determined before treatment between the three groups. After treatment, proteinuria was significantly higher in the vehicle treated mice than in mice treated with the ethyl acetate (EA) extract (**p < 0.0026, EA_low _versus vehicle; ****p < 0.0001, EA_high _versus vehicle).

All mice at age 28 weeks exhibited significant proteinuria (30 mg/dl or higher) before starting the treatment course. At this time, the mean proteinuria for the vehicle, EA_low _and EA_high _groups was 100.93 mg/dl, 121.98 mg/dl and 95.91 mg/dl, respectively (Fig 1a). Notably, during the treatment course, proteinuria increased in the mice treated with vehicle, with a mean proteinuria of 300 mg/dl at the age of 36 weeks. Of 14 animals in the vehicle group, 13 (93%) developed severe proteinuria (>500 mg/dl) before the end of the study (age of 42 weeks). Only one mouse in the vehicle group continued to maintain proteinuria below 100 mg/dl at the end of study. In contrast, 12 mice (80%) from the EA_high _group had 30 to 100 mg/dl of proteinuria at the start of treatment. In 9 out of the 15 animals, proteinuria was improved or maintained at the same low level (30 to 100 mg/dl) throughout the study. Proteinuria worsened in only four animals. In two of the four mice, worsening of proteinuria was noted two and four weeks after starting treatment. Two mice developed severe proteinuria before starting treatment that was unchanged during the treatment course. Similar to the EA_high _group, proteinuria was improved or maintained at a low level throughout the 14 week treatment in 7 out of 15 mice in the EA_low _group. In four animals from the EA_low _group, proteinuria was maintained at a low level and increased only at the very end of the treatment course. Four mice from the EA_low _group had severe proteinuria in the beginning of treatment without improvement. Figure 1b shows the comparison of proteinuria between groups before and after treatment. There was no significant difference in mean proteinuria between the three groups at the beginning of treatment. At the end of the treatment course, however, mean proteinuria was 496 mg/dl, 204 mg/dl and 190 mg/dl for the vehicle, EA_low _and EA_high _groups, respectively (*p *< 0.005, vehicle versus EA_low_; *p *< 0.0001, vehicle versus EA_high_).

**Figure 2:**
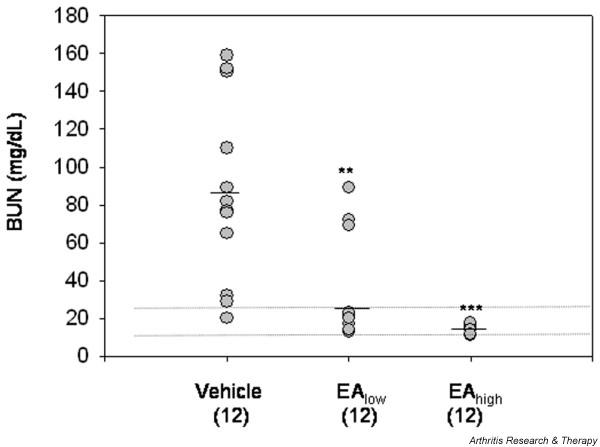
Renal function (blood urea nitrogen (BUN)) of the NZB/WF1 mice after treatment with vehicle or the ethyl acetate (EA) extract of *Tripterygium wilfordii *Hook F. Serum was collected from the animals for assay of BUN before euthanasia. Dotted lines show the normal range for BUN (17 to 28 mg/dl). Numbers in parentheses indicate the animals examined. The horizontal bars indicate the median values of each group. **p < 0.001, vehicle versus EA_low_; ***p < 0.01, vehicle versus EA_high_.

BUN was also determined and was increased and correlated with the severity of proteinuria in the three groups. As shown in Figure 2, 10 out of 12 tested animals from the vehicle group had increased BUN ranging from 32 mg/dl to 159 mg/dl (normal range <27 mg/dl). In contrast, in 9 out of 12 mice from the EA_low _group, BUN was within normal range and none of the mice in the EA_high _group exhibited elevated BUN. The mean BUN was 86.75, 29.73 and 19.09 mg/dl for the vehicle, EA_low _and EA_high _groups, respectively (*p *< 0.001).

### Effect of the EA extract treatment on kidney pathology

**Figure 3:**
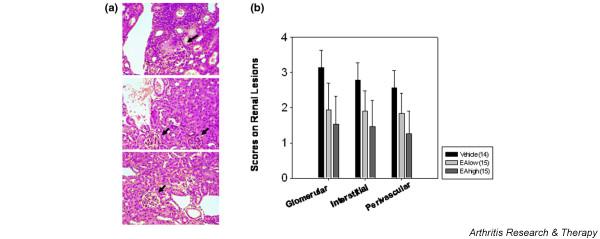
Changes in renal pathology as a result of treatment with the ethyl acetate (EA) extract of *Tripterygium wilfordii *Hook F. **(a) **Kidney from the vehicle group showed glomerulonephritis, tubular dilation and atrophy, and heavy cell infiltration in perivascular and interstitial region. Kidney sections from the mice of the **(b) **EA_low _and **(c) **EA_high _groups showed normal glomeruli and tubules. Slight cell infiltration in perivascular regions was seen in **(b)**. Results shown are representative of 14 animals of each group. Arrows indicate representative glomeruli. **(d) **Glomerular, interstitial and perivascular disease was scored from (0) to (4+) as described in Materials and methods. Numbers of mice examined are indicated in parentheses. *p < 0.05, **p < 0.01, ***p < 0.001, EA_low _or EA_high _versus vehicle. Original magnification × 400.

At the end of the study, kidneys obtained from 14 animals from the vehicle group and 15 from each of the EA treated groups were examined for histological changes. All kidneys from the vehicle group had glomerular, interstitial and vascular lesions (Figure 3). Glomeruli were the most severely affected. Glomerular lesions ranged from proliferative glomerulonephritis with influx of mononuclear cells and rare neutrophils to diffuse glomerular sclerosis with crescent formation, fibrinoid necrosis and proteinuria. Interstitial disease consisted of infiltration of mononuclear cells around tubules with tubular atrophy, dilation and thickened tubular basement membranes. Occasional tubules had necrotic tubular epithelium. Perivascular disease consisted of mononuclear cell infiltrates in a follicular pattern around large arteries and interlobular arteries. In the most severely affected mice, the mononuclear cells around blood vessels were confluent with interstitial inflammation. After 14 weeks of treatment, most mice treated with either the low or high dose of the EA extract had less severe kidney disease with significantly diminished glomerular, interstitial and perivascular lesions.

Figure 3d shows the comparison of the renal lesion scores between the three groups. Glomerular, interstitial and perivascular lesions were significantly less severe in the mice treated with the EA extract than those treated with vehicle. Most significant was the differences in glomerular lesions between the groups; *p *< 0.01, EA_low _versus vehicle; *p *< 0.001, EA_high _versus vehicle.

### Treatment with the EA extract limits lymphoid cell infiltration and immune complex deposition in kidneys

**Figure 4:**
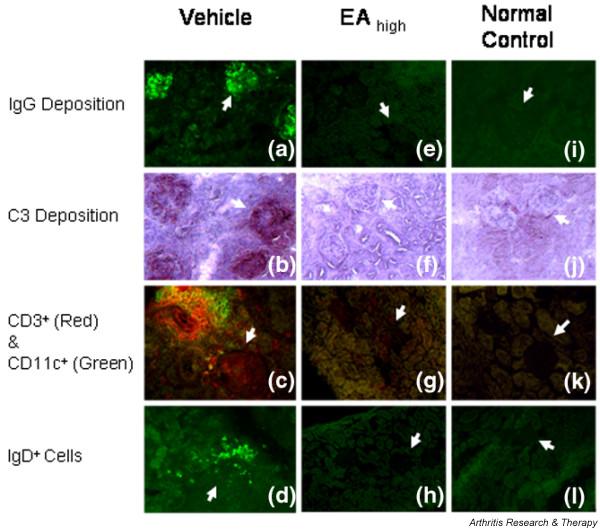
Immunohistochemical analysis of the kidney of NZB/WF1 mice after treatment with **(a-d) **vehicle or **(e-h) **the ethyl acetate (EA) extract of *Tripterygium wilfordii *Hook F. Kidney was examined for deposition of **(a,e,i) **IgG, (b,f,j) complement 3 (C3) and infiltration of **(c,g,k) **CD3^+ ^cells (in red) and CD11c^+ ^cells (in green) and (d,h,l) IGD^+ ^cells. Results shown are representative of five mice per group. Arrows indicate representative glomeruli.

IgG and C3 staining was observed only in glomeruli and the intensity of IgG and C3 was correlated with the severity of proteinuria (Fig. 4a,b,e,f). Remarkable deposition of both IgG and C3 was observed in the kidneys from all mice of the vehicle group. In contrast, significantly less or no glomerular deposition of either IgG or C3 was noted in the mice treated with either the low or high dose of the EA extract, similar to the normal control mice (Figure 4i,j).

Immunofluorescent staining was carried out to determine the impact of treatment on mononuclear cells infiltrating the kidneys. Both the extent and the density of T cells, B cells and macrophages/dendritic cells infiltrating the kidneys were related to the severity of proteinuria. Significant numbers of CD3^+ ^T cells, CD11c^+ ^myeloid cells, and IgD^+ ^B cells were found in the area around vessels that extended into the interstitium in the kidneys from the mice of the vehicle group (Figure 4c,d,g,h). Similar to that noted with normal C57BL/6j mice, there was almost no cellular infiltration in the kidneys from either the EA_low _or the EA_high _group.

### Changes in serum levels of anti-dsDNA antibody

**Figure 5:**
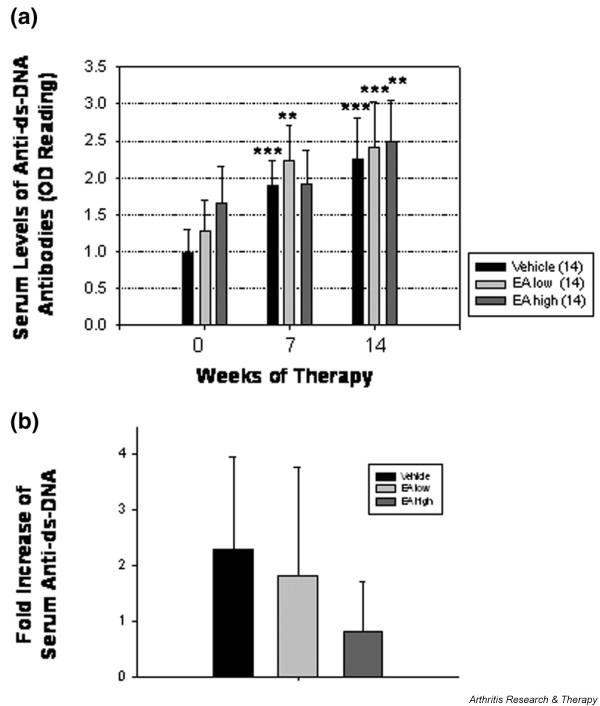
Serum anti-double-stranded (ds)DNA antibody in the NZB/WF1 mice before and after treatment with vehicle or the ethyl acetate (EA) extract of *Tripterygium wilfordii *Hook F. **(a) **Serum was collected before, 7 and 14 weeks after starting treatment. Anti-dsDNA antibody was determined by ELISA as described in Materials and methods. The mean of relative titers of anti-dsDNA antibody of serum obtained from normal C57BL/6j mice was 0.23 (0.1 to 0.3). Numbers in parentheses indicate the mice evaluated. Data are the mean ± standard error of the mean of the optical density (OD) readings on 1:20 diluted serum of each group. **p < 0.01, ***p < 0.001 at time 0 versus at 7 weeks or 14 weeks of treatment for the same group. **(b) **The same data were analyzed as the change in anti-dsDNA antibody titers from the baseline at 28 weeks of age. Original magnification × 400.

Autoantibody against dsDNA was examined before, 7 weeks and 14 weeks after starting treatment. Compared to normal C57BL/6j mice, sera from the NZB/WF1 mice of the three groups contained higher titers of anti-dsDNA antibody at 28 weeks of age. The relative titers of anti-dsDNA at this time for C57BL/6j mice ranged from 0.1 to 0.3, with an average of 0.23. All NZB/W F1 mice employed in the study exhibited elevated titers of this autoantibody, with averages of 1.0, 1.3 and 1.6 for the vehicle, EA_low _and EA_high_groups, respectively (Figure 5a). The levels of the anti-dsDNA autoantibody increased during the treatment course for all of the three groups. The increase of anti-dsDNA antibody at the end of treatment compared to that before treatment was statistically significant for the vehicle group (*p *< 0.001) and both the EA_low _(*p *< 0.001) and EA_high _(*p *< 0.01) groups. Moreover, there was no significant difference in the mean titers of this autoantibody between groups, either before or after treatment.

Since the initial titer of anti-DNA antibody was higher in the mice treated with the high dose of the EA extract, the fold increases in this antibody upon completion of the treatment course were compared between the three groups. The mean fold increases of anti-dsDNA antibody were 2.29, 1.81 and 0.81 for vehicle, EA_low _and EA_high _groups, respectively (Figure 5b). Compared to the vehicle group, mice treated with the high dose of the EA extract had less increase in serum anti-dsDNA antibody, although this did not reach statistical significance (*p *= 0.07).

### Effect of treatment with the EA extract on spleen lymphoid cells

**Figure 6:**
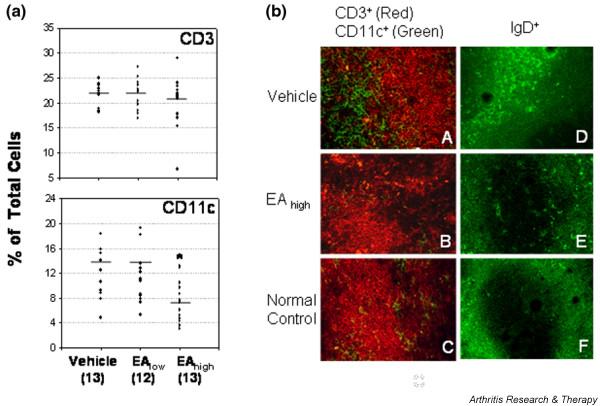
Changes in splenic mononuclear cells as a result of treatment with the ethyl acetate (EA) extract of *Tripterygium wilfordii *Hook F (TwHF). **(a) **Flow cytometric analysis of spleen mononuclear cells from NZB/WF1 mice after treatment with vehicle or the EA extract of TwHF. Horizontal bars indicate the median value for each group. Numbers of mice analyzed are indicated in parentheses. *p < 0.05, EA_high _versus vehicle. **(b) **Immunohistochemical analysis of spleen from NZB/WF1 mice after treatment with vehicle (A,C) or the EA extract of TwHF (B,D). CD3^+ ^cells (red) and CD11c^+ ^cells (green) are shown in (A,B,C) and IgD^+ ^cells shown in (D,E,F). Results shown are representative of five mice per group. Original magnification × 400.

Splenomegaly was observed in most NZB/WF1 mice of the vehicle group. Mice treated with the EA extract had significantly smaller spleens than those treated with vehicle. To examine the effect of the EA extract in greater detail, spleen mononuclear cells were analyzed by flow cytometry. There was no significant difference in the percentage of CD3^+ ^T cells over the total cells between the NZB/W F1 mice of the three groups (Figure 6a). The ratio of CD11c^+ ^myeloid cells to the total cells, however, was significantly lower in the NZB/W F1 mice treated with the higher dose of the EA extract than in those treated with vehicle (*p *< 0.01).

To confirm the information obtained from flow cytometry, fluorescent staining of spleen cryosections was carried out. Very large follicles with abundant IgD^+ ^B cells, CD3^+ ^T cells as well as CD11c^+ ^myeloid cells forming a greatly increased white pulp were observed in the spleens from the mice of the vehicle group (Fig. 6b). In contrast, spleens of mice treated with the EA extract exhibited smaller follicles with significantly less numbers of IgD^+ ^B cells. In addition, CD11c^+ ^myeloid cells were only infrequently observed in the spleens of the mice treated with the EA extract, consistent with the results from flow cytometry. The densities of follicles and CD3^+ ^T cells in the spleens of the EA treated mice were not significantly different from those in the vehicle treated mice. The profile of cells observed in the spleens of the EA extract treated mice was very similar to that of the normal C57BL/6j mice.

## Discussion

In this study, we selected NZB/W F1 mice to examine the potential therapeutic role of the EA extract of TwHF in SLE. The study was designed to start therapy of the NZB/W F1 mice at the age of 28 weeks because all mice at this time had positive anti-dsDNA antibody titers and more than 90% of the mice had detectable proteinuria, indicating that autoimmune nephritis was established before the start of treatment in these mice. Results of the study show that 93% of mice treated with vehicle had progressive glomerulonephritis, documented by severe proteinuria, elevated BUN and histological abnormalities at the end of the study. In contrast, proteinuria and pathological changes in the kidneys were significantly improved or maintained at mild levels of disease in most mice treated with the EA extract, suggesting that the EA extract exerted a therapeutic effect on established lupus nephritis in NZB/W F1 mice. This result is important because therapy of human SLE involves treatment of established disease. Therapeutic trials in NZB/W F1 mice frequently involve initiation of therapy before disease onset [16,26,28]. Only a few agents have demonstrated benefit after the onset of disease [15-17]. The finding that the EA extract of TwHF is effective as a therapy for established NZB/W F1 murine lupus suggests that its efficacy may be more easily translated to treatment of human lupus.

It is important to note that the current results were obtained with a standardized extract of TwHF. TwHF has been widely used for several decades in China in the treatment of a variety of autoimmune disorders, including RA and, to a lesser extent, SLE. However, the extracts employed in these studies were manufactured from wild TwHF plants collected from varying locations. As the composition of these preparations was uncertain, the treatment dose of the extract was determined according to the weight of the raw plant materials and the basis for the clinical effects could not be determined. Since there was no quantitative standard for these TwHF preparations, reproducible clinical efficacy and toxicity could not be ensured. The EA extract of TwHF employed in the present study was prepared from plant material characterized by HPLC and liquid chromatography-mass spectroscopy and by a standardized manufacturing protocol with quantitative control of the major active components, triptolide and tripdiolide, and their ratios [21]. The EA extract of TwHF was also quantitatively evaluated and standardized for its *in vitro *bioactivities and *in vivo *toxicity before being applied to the current studies [24]. It is important to note that the extract employed in the current study has already been tested in phase I and phase II clinical trials in patients with RA and has been found to be both safe and effective [19,20].

TwHF has been tested for its effect on the MLR-*lpr*/*lpr *lupus model [11,12]; however, the preparations used in these two studies were poorly standardized. In addition, treatment was started before the development of autoimmune disease [11]. Notably, the results of the two studies differed in that in one [12] no effect on renal histology was noted whereas improvement was noted in the other [11]. This could be related to the use of different extracts of TwHF, both of which were poorly characterized. In the current study, a well characterized extract was employed in a therapeutic approach in animals with established disease; histological changes were well correlated with clinical improvement of the kidney disease and the extract showed considerable efficacy. Previously, treatment of MRL-*lpr*/*lpr *mice with an extract of TwHF after onset of disease resulted in improvement in proteinuria but not renal histopathology [11]. Whether this related to differences in the potency of the extract employed or differences in the murine models is unknown.

The mechanism by which the EA extract reduced autoimmune nephritis in the NZB/W F1 mice could relate to the immunosuppressive effect of its active components. The immunosuppressive effects of the extract of TwHF have been documented in both *in vitro *and *in vivo *studies. *In vitro*, the extract of TwHF inhibited proliferation and IL-2 and interferon-γ production by T cells in response to antigen and mitogen stimulation [23]. Inhibition of IL-2 production reflected an inhibition of IL-2 gene transcription [25]. In agreement with the results of *in vitro *studies, IL-2 production by spleen cells of mice treated with the EA extract was less than that from control animals [29]. TwHF also suppressed humoral responses. *In vitro*, the extract of TwHF inhibited proliferation and production of antibody by purified human B cells in response to stimulation with polyclonal B cell activators, indicating that triptolide and tripdiolide directly affected the function of B cells as potently as that of T cells [23]. *In vivo *treatment with TwHF or triptolide has also been reported to inhibit antibodies against sheep red blood cells (SRBC) in mice [30]. In addition, we have found that treatment of normal mice with the EA extract of TwHF blocked the induction of the primary antibody responses to immunization with trinitrophenyl conjugated keyhole limpet hemocyanin (TNP-KLH). However, delaying treatment until 30 days after immunization when antibody titers were already elevated resulted in no inhibitory effect (unpublished data). These results suggest that the active components of the EA extract of TwHF inhibited the initiation of antibody responses but not ongoing antibody production by established plasma cells. Notably, treatment of RA patients with an extract of TwHF significantly reduced the production of IgM and IgM-rheumatoid factor by non-stimulated or pokeweed mitogen-stimulated peripheral blood mononuclear cells isolated from treated patients [31]. These findings are all consistent with the possibility that the EA extract of TwHF reduced autoimmune nephritis in NZB/W mice and normalized splenic architecture by a direct immunosuppressive action.

Some of the effects of the extract could also have related to an anti-inflammatory effect of its active components. A direct anti-inflammatory effect has been demonstrated by the finding that the EA extracts or purified components of TwHF inhibited *in vitro *production of many inflammatory mediators, including prostaglandin E_2 _(PGE_2_)and nitric oxide, and inhibited transcription of relevant molecules, such as cyclooxygenase 2 and inducible nitric oxide synthase [29,32,33]. Treatment of animals with extracts of TwHF also significantly suppressed production of IL-6, tumor necrosis factor, PGE_2 _and nitric oxide by cultured spleen mononuclear cells from these animals [29,34]. These findings suggest that some of the benefit of the EA extract of TwHF could relate to the anti-inflammatory effects of its components. The combination of the anti-inflammatory and immunosuppressive actions of the active components of TwHF could explain its effectiveness in the treatment of nephritis in the NZB/W F1 model of lupus.

The current study shows remarkable elimination of glomerular deposition of IgG and C3 but no apparent effect on circulating levels of anti-dsDNA antibodies in the EA treated mice. It is notable that many other reagents that have been reported to be effective in lupus nephritis in NZB/W F1 mice have not necessarily had an effect on anti-dsDNA titers, depending on the timing of therapy. For example, treatment of NZB/W F1 mice with cyclophosphamide, prednisone or azathioprine has been reported to reduce serum anti-dsDNA antibody when drug administration was started before appearance of proteinuria and anti-nuclear antibodies [16]. Similarly, NZB/W F1 mice treated with cyclosporine from the age of 7 months showed decreased serum levels of anti-dsDNA. However, anti-dsDNA antibody levels were unaffected in mice treated from the age of 8 months [35].

The mechanism for the decrease of kidney deposition of IgG and C3 in the EA treated NZB/W F1 mice is uncertain, since there was no significant reduction of serum anti-dsDNA antibody titers in these mice. It is possible that pathogenic anti-dsDNA antibodies may have been produced locally within the kidney and not be reflective of serum anti-dsDNA levels. In this regard, plasma cells have been noted in the kidney of NZB/W F1 mice [36]. Treatment with the EA extract of TwHF could have suppressed the production of these locally secreted autoantibodies but not those derived from long lived plasma cells in the bone marrow. This is consistent with the findings that EA extract treatment of NZB/W F1 mice completely eliminated the cellular infiltrate in the kidney. Second, it is possible that glomerular deposition was accounted for by autoantibodies other than anti-dsDNA, explaining the dichotomy between serum anti-dsDNA and glomerular deposition of IgG. In this regard, a variety of autoantibodies have been eluted from murine lupus kidneys [37] and proliferative nephritis has been noted in the absence of anti-dsDNA antibody in some murine models of SLE [38-40]. Finally, the EA extract may have changed the micro-environment of the basement membrane of the glomeruli and thereby altered IgG deposition.

Besides the effects on glomerular deposition of IgG and C3, treatment with the EA extract of TwHF also had a major impact on the hypercellularity within the kidney and spleen. Although the effect in the kidney could be secondary to the decrease in immune complex deposition, it is possible that the impact on hypercellularity in both kidney and spleen are related to direct immunosuppressive activities of the active components of TwHF. The effects were most striking on CD11c+ myeloid cells and IgD+ B cells. The active components of TwHF have been shown to have several immunosuppressive effects, including inhibition of the transcription of cytokine genes and genes encoding other molecules involved in the production of inflammatory mediators. The effect of the active components of TwHF can be explained by their capacity to inhibit the activity of transcription factors, such as NF-κB, the activator protein 1 (AP-1), the nuclear factor of activated T cells (NFAT) and the Octamer transcription factor 1 (Oct-1) [41-43]. Whether the active components of the EA extract of TwHF are exerting a direct effect on lymphoid and myeloid cells in the NZB/W F1 mouse or an indirect effect by inhibiting the production of cytokines and/or inflammatory mediators is currently unknown. Regardless, it is important to note that therapy with the EA extract of TwHF resulted in the loss of splenomegaly with a normalization of splenic architecture. There was no evidence, however, of pathological immunodepletion in either the NZB/W F1 mice or in humans. Rather, therapy appeared to restore immunological homeostasis. Regardless of the precise mechanism, it is clear that treatment with the EA extract of TwHF stabilized renal function and improved renal pathology in NZB/W F1 mice.

## Conclusion

The EA extract of TwHF effectively controlled and reduced autoimmune nephritis in NZB/W F1 mice and, therefore, may provide a novel therapeutic approach in SLE patients.

## Abbreviations

BSA = bovine serum albumin; BUN = blood urea nitrogen; C3 = complement 3; ds = double stranded; EA = ethyl acetate; FITC = fluorescein isothiocyanate; HPLC = high-performance liquid chromatography; IL = interleukin; LD50 = lethal dose 50%; RA = rheumatoid arthritis; SLE = systemic lupus erythematosus; TwHF = *Tripterygium wilfordii *Hook F.

## Competing interests

The authors declare that they have no competing interests.

## Authors' contributions

XT participated in the design of the study, performed the experimental assays and wrote the manuscript. FF participated in urine analysis and immunohistochemical assays. VH performed pathological studies and wrote the manuscript. NL participated in pathological studies and was involved in drafting the manuscript. PL participated in the design of the studies and was involved in drafting the manuscript and approval of the version to be published.

## Figures and Tables

**Table 1:** Grading of kidney lesions

Score	Glomerulonephritis	Interstitial nephritis	Vessels
1+	Focal, mild or early proliferative	1–3 small foci (5–10 cells) of MNC *	MNC around renal pelvis/blood vessels
2+	Multifocal proliferative with increased matrix and inflammatory cells	Mild MNC infiltrates around individual tubules; isolated atrophied tubules	MNC infiltrates around main arteries; small foci 10–20 cells of MNC around interlobular arteries
3+	Diffuse proliferative	More extensive infiltrates with large foci of tubular atrophy	Individual foci of MNC around small arterial branches more extensive
4+	Extensive sclerosis/ crescents: proteinuria	Extensive MNC infiltrates between tubules; extensive tubule atrophy/necrosis	MNC infiltrates extend into surrounding parenchyma/most vessels affected/vasculitis

*MNC = mononuclear cells.

## References

[B1] Lipsky PE, Tao XL (1997). A potential new treatment for rheumatoid arthritis: Thunder God Vine. Semin Arthritis Rheum.

[B2] Tao XL, Lipsky PE (2000). The Chinese anti inflammatory and immunosuppressive herbal remedy *Tripterygium wilfordii* Hook F. Rheum Dis Clin North America.

[B3] Wang QW, Li LS, Zhang JH (1991). Clinical studies of treatment of idiopathic IgA nephropathy with *Tripterygium wilfordii* Hook f. Jiang Su Yi Yao.

[B4] Li H, Cheng QR, Dong DC (1993). Observation on clinical effect on treatment of IgA nephropathy with *Tripterygium wilfordii* Hook. Shanghai Yi Xue.

[B5] Peng SY, Guo SJ, Dong SR (1983). Treatment of Henoch-Shoenlein purpura with total glycosides of *Tripterygium wilfordii*. Jiang Su Yi Yao.

[B6] Pan YR (1987). Treatment of purpura nephritis with *Tripterygium wilfordii* Hook F. Acta Acad Med Sinicae.

[B7] Liao WQ (1980). Observations on the therapeutic effect of *Tripterygium wilfordii* Hook f on systemic lupus ethythematosus. Pi Fu Beng Fang Zhi Yan Jiou Tong Xun.

[B8] Qin WZ, Yang SM, Zhu GD, Liu CH, Li F, Feng SF, Han KY, Tang GT, Gong ZM, Wang HT (1982). Treating 103 cases of SLE with *Tripterygium wilfordii* Hook f. Chin J Dermatol.

[B9] Li ZM, Zheng CS (1982). Observation on treatment of systemic lupus erythematosus with extract of *Tripterygium wilfordii* Hook. Xin Zhong Yi.

[B10] Xue GY, Xue JH, Tang YC, Fan ZH (1984). Treatment of 21 patients with SLE with *Tripterygium wilfordii* Hook. Zhong Hua Pi Fu Ko Za Zhi.

[B11] Gu WZ, Banerjee S, Rauch J, Brandwein SR (1992). Suppression of renal disease and arthritis, and prolongation of survival in MRL-Apr mice treated with an extract of *Tripterygium wilfordii* Hook f. Arthritis Rheum.

[B12] Zhang XY, Tsuchiya N, Dohi M, Yamamoto K, Okadaira HK, Miyamoto T (1992). Prolonged survival of MRL-lpr/Ipr mice treated with *Tripterygium wilfordii* Hook f. Clin Immun Immunopathol.

[B13] Andrews BS, Eisenberg RA, Theoflopous AN, Izui S, Wilson CB, McConahey ED, Muphy ED, Roths JB, Dixon RJ (1978). Spontaneous murine lupus-like syndromes. Clinical and immunopathological manifestations in several strains. J Exp Med.

[B14] Hahn B, Qallace HB (1997). Dubois' lupus erythematosus. Animal Models of Systemic Lupus Erythematosus.

[B15] Borel Y, Lewis RM, Andre-Schwatz J, Stollar BD, Diener E (1978). Treatment of lupus nephritis in adult(NZB+NZW)F1 mice by cortisone-facilitated tolerance to nucleic acid antigens. J Clin Invest.

[B16] Hahn BH, Knotts L, Hamilton TR (1975). Influence of cyclophosphamide and other immunosuppressive drugs on immune disorders and neoplasia in NZB/NZW mice. Arthritis Rheum.

[B17] Schiffer L, Sinha J, Wang X, Huang W, Gonsdroff GV, Schiffer M, Madaio MP, Davidson A (1997). Short term administration of costimulatory blockade and cyclophosphamide induces remission of systemic lupus erythematosus nephritis in NZB/W F1 mice by a mechanism downstream of renal immune complex deposition. J Immunol.

[B18] Wakeland EK, Liu K, Graham RR, Behrens TW (2001). Delineating the genetic basis of systemic lupus erythematosus. Immunity.

[B19] Tao XL, Cush JJ, Garret MM, Lipsky PE (2001). A phase I study of the ethyl acetate extract of the Chinese anti-rheumatic herb, *Tripterygium wilfordii* Hook F in rheumatoid arthritis. J Rheumatol.

[B20] Tao XL, Younger J, Wang B, Lipsky PE (2002). Benefit of an extract of *Tripterygium wilfordii* Hook F in patients with rheumatoid arthritis: a double-blind, placebo-controlled study. Arthritis Rheum.

[B21] Cai Ji, Tao XL, Lipsky PE (1994). High performance liquid chromatography determination of triptolide and triptolide in ethyl acetate extract of Tripterygium wilfordii Hoo.F. J Liquid Chromatography.

[B22] Li LZ, Chen SF, Wang FJl (1982). Pharmacological studies of the ethyl acetate extract of Tripterygium wilfordii Hook f. Zhong Cao Yao.

[B23] Tao XL, Davis LS, Lipsky PE (1991). Effect of an extract of the Chinese herbal remedy, Tripterygium wilfordii Hook f on human immune responsiveness. Arthritis Rheum.

[B24] Tao XL, Cai JJ, Lipsky PE (1995). The identity of immunosuppressive components of the ethyl acetate extract and chloroform methanol extract (T)_2 _of Tripterygium wilfordii Hook. f. J Parmacol Exp Ther.

[B25] Tao XL, Davis LS, Hashimoto K, Lipsky PE (1996). The Chinese herbal remedy, T _2_, inhibits mitogen-induced cytokine gene transcription by T cells, but not initial signal transaction. J Parmacol Exp Ther.

[B26] Macanovic M, Sinicrop D, Shak S, Baughman S, Thiru S, Lachmann PJ (1996). The treatment of systemic lupus erythematosus (SLE) in NZB/W F1 hybrid mice; studies with recombinant murine DNase and with dexamethasone. Clin Exp Immunol.

[B27] Chan OTM, Hannum LG, Haberman AM, Madaio MP, Shlomchik MJ (1999). A novel mouse with B cells but lacking serum antibody reveals an antibody-independent role for B cells in murine lupus. J Exp Med.

[B28] Ramos MA, Pinera C, Setien MA, Buelta L, De Cos MA, Francisco ALM, Merino R, Arias M (2003). Modulation of autoantibody production by mycophenolate mofetil: effect on the development of SLE in (NZBxNZW)F1 mice. Nephrol Dial Transplant.

[B29] Tao XL, Ma L, Mao YP, Lipsky PE. (1999). Suppression of carrageenan-induced inflammation *in vivo* by an extract of the Chinese herbal remedy, *Tripterygium wilfordii* Hook. Inflamm Res.

[B30] Li LZ, Chen SF, Wang (1986). Effect of triptolide on inflammation and immune function. Zhong Guo Yao Li Xue Tong Bao.

[B31] Tao XL, Shi YP, Cheng XH, Zhang NZ (1988). Mechanism of treating rheumatoid arthritis with Tripterygium wilfordii Hook. I. Effect on Secretion of total IgM and IgM-RF by peripheral blood mononuclear cells (PBMC). Acta Acad Med Sinicae.

[B32] Tao XL, Schulze-koops H, Ma L, Cai J, Mao YP, Lipsky PE (1998). Extract of *Tripterygium wilfordii* Hook. F inhibit induction of cyclooxygenase-2 activity and PGE_2 _production. Arthritis Rheum.

[B33] Wang B, Ma L, Tao XL, Lipsky PE (2004). Triptolide, an active component of the Chinese herbal remedy *Tripterygium wilfordii* Hook F, inhibits production of nitric oxide by decreasing inducible nitric oxide synthase gene transcription. Arthritis Rheum.

[B34] Tao XL, MA L, Cai J (1996). Treatment with an ethyl acetate extract of *Tripterygium wilfordii* Hook f improves joint inflammation in HLA B27 transgenic rats. (60th) National Scientific Meeting of American College of Rheumatology. Orlando, Florida. Arthritis Rheum.

[B35] Okudaira H, Terada E, Okudaira K (1987). Animal models utilize in the research of autoimmune disease control: experimental therapy of glomerulaonephritis in NZB/W F1 mice. Prog Clin Biol Res.

[B36] Cassese G, de Lindenau S, Boer B, Arce S, Hauser A, Riemekasten G, Berek C, Hiepe F, Krenn V, Radbruch A (2001). Inflamed kidneys of NZB/W mice are a major site for the homeostasis of plasma cells. Eur J Immunol.

[B37] Dixon FJ, Lidstone MBA, Tonietti G (1971). Pathogenesis immune complex glomerulonephritis of New Zealand mice. J Exp Med.

[B38] Chan OTM, Hannum LG, Haberman AM, Madaio MP, Shlomchik MJ (1999). A novel mouse with B cells but lacking serum antibody reveals an antibody-independent role for B cells in murine lupus. J Exp Med.

[B39] Waters ST, Mcduffie M, Bagavant H, Deshmukh US, Gaskin F, Jiang C, Tung K, Fu SM (2004). Breaking tolerance to double stranded DNA, Nucleosome, and other nuclear antigens is not required for the pathogenesis of lupus glomerulonephritis. J Exp Med.

[B40] Christensen SR, Kashgarian M, Alexopoulou L, Flavell RA, Akira S, Shlomchik MJ (2005). Toll-like receptor 9 control anti-DNA autoantibody production in murine lupus. J Exp Med.

[B41] Kim YH, Lee SH, Lee JY, Choi SW, Park JW, Kwon TK (2004). Triptolide inhibits murine-inducible nitric oxide synthase expression by down-regulating lipopolysaccharide-induced activity of nuclear factor-kappa B and c-Jun NH2-terminal kinase. Eur J Pharmacol.

[B42] Zhuang WJ, Fong CC, Cao J, Ao L, Leung CH, Cheung HY, Xiao PG, Fong WF, Yang MS (2004). Involvement of NF-kappaB and c-myc signaling pathways in the apoptosis of HL-60 cells induced by alkaloids of Tripterygium hypoglaucum (levl.) Hutch. Phytomedicine.

[B43] Jiang XH, Wong BC, Lin MC, Zhu GH, Kung HF, Jiang SH, Yang D, Lam SK (2001). Functional p53 is required for triptolide-induced apoptosis and AP-1 and nuclear factor-kappaB activation in gastric cancer cells. Oncogene.

